# Comparing direct oral anticoagulants and vitamin K antagonist use in morbidly obese patients with venous thromboembolism: A single center retrospective cohort study

**DOI:** 10.1002/jha2.418

**Published:** 2022-03-24

**Authors:** Lia C. Scott, Juan Li, Lorraine A. Cafuir, Manila Gaddh, Christine L Kempton

**Affiliations:** ^1^ Department of Hematology and Medical Oncology Emory University School of Medicine Atlanta Georgia USA

**Keywords:** body mass index, dabigatran, factor Xa inhibitors, proportional hazards models, recurrence, venous thromboembolism, warfarin

## Abstract

**Introduction**: Limited data exists on the safety and efficacy of direct‐acting oral anticoagulants (DOAC) use in morbidly obese patients with venous thromboembolism (VTE). Given the benefits of DOAC use over vitamin K antagonists (VKAs), in terms of monitoring requirements, and dietary and drug interactions, it is important to evaluate whether this is consistent in the higher risk for VTE recurrence morbidly obese group body mass index (BMI ≥ 40 kg/m^2^).

**Materials and methods**: This retrospective, single‐center cohort study included patients with a BMI of at least 40 kg/m^2^ who were admitted to Emory University Hospital from 1^st^ January 2012 to 31^st^ May 2020 with acute VTE, and subsequently initiated on anticoagulation treatment with either DOAC or VKA (warfarin). Univariate and bivariate analyses were used to evaluate differences in demographics by treatment type and BMI. Multivariate Cox proportional hazard regression was used to assess the risk of VTE recurrence by type of treatment among morbidly obese patient subgroup.

**Results**: There were 247 (11.8%) morbidly obese (≥ 40 kg/m^2^) patients who were more likely than non‐obese patients to be younger, female, and of non‐white race. Thirty percent of the study population (n=74) had a BMI >50 kg/m^2^. T ime‐to‐event analysis confirmed that the hazard of experiencing a recurrent thrombosis was not statistically significantly different among morbidly obese patients treated with a DOAC compared with VKA (hazard ratio [HR]: 0.28, confidence interval [CI] 0.07‐1.11, *p *= 0.07).

**Conclusions**: This study aligns with previous literature and confirms that morbidly obese patients receiving DOAC or VKA have similar risks of recurrent VTE.

## 1 INTRODUCTION

Direct oral anticoagulants (DOACs) have been approved for the treatment of venous thromboembolism (VTE) has replaced vitamin K antagonists (VKAs) as the standard of care. Compared with VKAs which have a narrow therapeutic window, need for monitoring, and have many food and drug interactions, DOACs offer many advantages including their ease of administration, fixed dosing, fast onset and offset of action, fewer drug and food interactions, lower bleeding risk, and the lack of need for routine monitoring [1, 2, 3, 4]. DOACs include dabigatran, rivaroxaban, apixaban, and edoxaban. Compared to VKAs, DOACs were equally effective in preventing recurrence, and are associated with less bleeding [4].

Obesity is associated with a greater risk of VTE and is a substantial public health problem in the United States with a prevalence of 42.4% in 2017–2018 among adults [5, 6, 7]. Unfortunately, the safety and efficacy of DOACs in obese patients is unclear as patients with weights > 120 kg were not included in the phase 3 licensing trials of these products [8]. Given that DOACs are associated with less bleeding and similar efficacy as VKA and that obesity increases the risk of VTE, it is important to evaluate the effectiveness of treatments within this group [4, 5, 6]. Observational data evaluating the efficacy and safety of DOACs in obese patients has been evolving through reports of real‐world and observational studies. A large study analyzing data from two US claims databases found that morbidly obese patients with VTE receiving rivaroxaban had similar risks of recurrent VTE and major bleeding compared with patients treated with warfarin [9]. Recent guidelines from the International Society of Hemostasis and Thrombosis suggested the DOACs can be safely used in those with a BMI > 40 kg/m^2^; however, as the body mass index (BMI) approaches or exceeds 50 kg/m^2^ the data supporting the use of DOAC thins [10]. The objective of this study is to quantify the difference in the risk of VTE recurrence in morbidly obese patients at 6 months, 1 year, and 5 years after acute VTE by type of treatment, hypothesizing that there will not be a statistically significant difference in risk of recurrent VTE in morbidly obese patients treated with DOAC or VKA.

## 2 METHODS

### 2.1 Study design and population

This retrospective single‐center cohort study analyzed data from Emory Healthcare Clinical Data Warehouse and included patients who were at least 18 years old when admitted at Emory University Hospital from 1^st^ January 2012 to 31^st^ May 2020. Inclusion criteria were as follows: (1) new acute VTE, based on the International Classification of Diseases Ninth and Tenth Revision Clinical Modification (ICD‐9/10‐CM) codes for pulmonary embolism (PE) (ICD‐10‐CM: I26.99; ICD‐9‐CM: 415.13, 415.19) and deep vein thrombosis (DVT) (ICD‐10‐CM: I81, I82.3, 182.4, I82.62, I82.90; G08. ICD‐9‐CM: 325, 416.2, 452.0, 453.0, 453.2, 453.3, 453.40‐42, 453.50‐52, 453.6, 453.74‐77, 453.79, 453.81‐87, 453.89); (2) had a radiographic study (ultrasound and/or computed tomography angiogram [CTA]) and; (3) started anticoagulation therapy with DOACs (rivaroxaban, apixaban, and dabigatran) or VKA, specifically warfarin, during that admission. Patients were excluded if there was no documented BMI at the start of anticoagulation (± 30 days) and investigators were unable to confirm if the patients had started anticoagulation therapy during the admission. Patients were followed from the initial acute VTE admission date until date of recurrent VTE, the date of discontinuation of anticoagulation as recorded in the electronic medical record (EMR) or the last encounter within the Emory Healthcare system. The study was reviewed and approved by the Emory University Institutional Review Board.

### 2.2 Data collection

All patients were characterized by BMI (super obesity: BMI ≥ 50 kg/m^2^; morbid obesity: BMI ≥ 40 kg/m^2^; nonmorbid obesity: BMI < 40 kg/m^2^), age at diagnosis, gender, and race. Gender was classified as male and female and race was classified as non‐white and white. Treatment was stratified by hospital anticoagulation therapy medication order: DOACs (rivaroxaban, apixaban, and dabigatran) or VKA. Comorbidities were quantified using the Charlson Comorbidity Index (CCI), a validated prognostic indicator for mortality that classifies comorbid conditions, where a score of 0 indicates no comorbidities. The study focused on patients with a BMI ≥ 40 kg/m^2^ [11, 12].

### 2.3 Outcome

A recurrent VTE was defined as readmission with a new VTE confirmed by ultrasound for DVT or CTA for PE and determined through individual review of clinical medical record. We confirmed a recurrent DVT and PE based on reading radiologist's report that interpreted the clots as new and true clots.

### 2.4 Statistical analysis

Descriptive statistics and tests for group differences were employed. Univariate analyses comparing anticoagulation treatment and BMI across all covariates were conducted. Continuous variables (age at diagnosis, BMI, CCI) were presented as means and standard deviations (SDs) or median and interquartile range (IQR) and compared using the Student *t*‐test or Wilcoxon rank sum test; categorical variables (gender, race) were presented as frequencies and percentages and compared using chi‐square tests.

Multivariate Cox proportional hazards models were developed to examine the relationship between 5‐year time to recurrent venous thrombosis and anticoagulation treatment with DOAC versus VKA use while controlling for patient characteristics. The proportional hazard assumption was tested for each variable by examining the log of negative log plots, and all covariates were expressed as adjusted hazard ratios (HRs) with corresponding 95% confidence interval (CI). The cumulative incidence function which accounts for competing risks was used to test the cumulative incidence of recurrent VTE at 6 months, 1 year, and 5 years, with the Gray test for differences by treatment type [13]. All analyses were conducted using SAS 9.4 (SAS Institute Inc., Cary, NC), and approval from institutional review board for this study was obtained from Emory University. Statistical significance was assessed at *p* value < 0.05 or 95% CI excluding 1.0.

## 3 RESULTS

**FIGURE 1:**
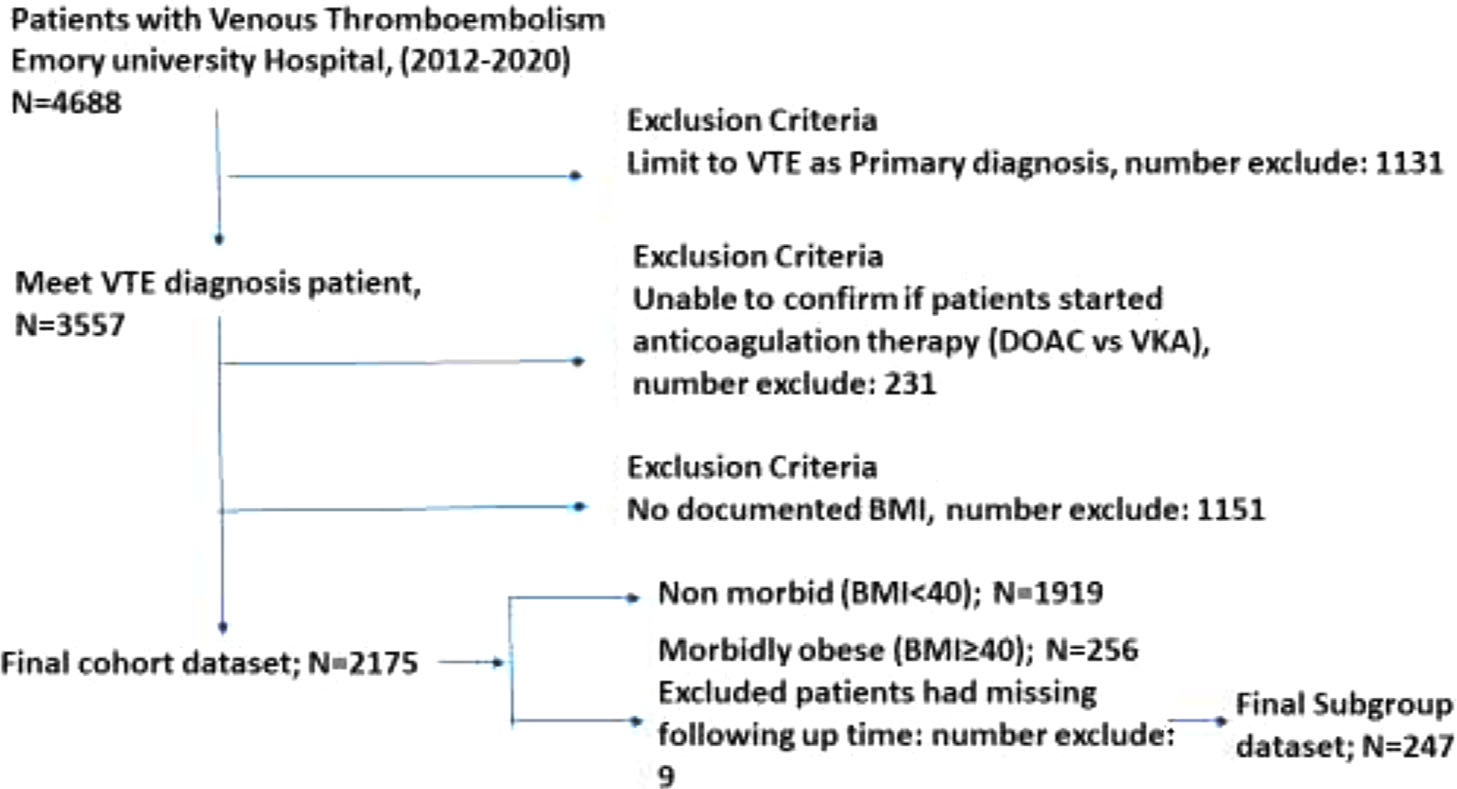
Flow diagram of study participants’ inclusion and exclusion criteria

Our entire cohort consisted of 2175 VTE patients, with 247 morbidly obese patients (Figure 1). Among patients, 118 patients (47.8%) had received VKAs and 129 (52.2%) received a DOAC. The mean age at diagnosis was 53.8 years (SD 14.2), and the mean BMI was 48.2 kg/m^2^ (SD 10.6). Women represented 68.4% of the cohort and 37.7% were of white race. There was no statistically significant difference in age, BMI, gender, race, and CCI between DOAC and VKA groups (Table 1). There were 76 patients (32.4%) with a BMI of 50 kg/m^2^ or greater. Morbidly obese patients were younger and more likely to be female and of non‐white race compared to non‐morbidly obese patients. Those with super obesity were similar to subjects with BMI ≥ 40 kg/m^2^ and < 50 kg/m^2^ except that they were more likely to be female.

**TABLE 1:** Descriptive statistics of patients with a body mass index (BMI) ≥ 40 grouped by treatment type and obesity status

	**Cases with morbid obesity (BMI ≥ 40 kg/m^2^)**				**Cases with super obesity (BMI ≥ 50 kg/m^2^)**			
**Characteristic**	**Total cases (*n* = 247)**	**DOAC (*n* = 129)**	**VKA (*n* = 118)**	***p* Value**	**Total cases (*n* = 76)**	**DOAC (*n* = 40)**	**VKA (*n* = 36)**	***p* Value**
Age at diagnosis, year, mean (SD)	53.8 (14.2)	55.4 (14.2)	52.1 (14.1)	0.066^a^	52.8 (11.9)	54.8 (11.2)	50.6 (12.5)	0.134^a^
BMI, mean (SD)	48.2 (10.6)	47.9 (11.4)	48.7 (9.8)	0.272^a^	58.1 (9.6)	56.5 (8.4)	59.9 (10.6)	0.094^a^
Gender, *n* (%)				0.306^b^				0.009^b^
Female	169 (68.4)	92 (71.3)	77 (65.3)		55 (72.4)	34 (85.0)	21 (58.3)	
Male	78 (31.6)	37 (28.7)	41 (34.7)		21 (27.6)	6 (15.0)	15 (41.7)	
Race, *n* (%)				0.499^b^				0.883^b^
Non‐white	154(62.3)	83 (63.4)	71 (60.1)		45 (59.2)	24 (60.0)	21 (58.3)	
White	93 (37.7)	46 (35.6)	47 (39.8)		31 (40.8)	16 (40.0)	15 (41.7)	
Charlson Comorbidity Index score, mean (SD)	1.85 (1.7)	1.93 (1.7)	1.77 (1.7)	0.438^a^	1.76 (1.6)	1.98 (1.3)	1.5 (1.8)	0.054^a^

Abbreviations: DOAC, direct acting anticoagulant; SD, standard deviation; VKA, vitamin K antagonist.

^a^ Student *t*‐test for age; Wilcoxon rank sum test for BMI, Charlson Comorbidity Index score.

^b^ Chi‐square test for treatment type, gender, and race.

**FIGURE 2:**
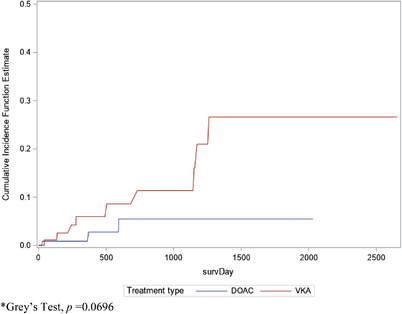
Cumulative incidence of recurrent venous thromboembolism (VTE) by treatment type among morbidly patients. *Grey's test, *p *= 0.0696

Nine patients were missing follow‐up time. The total follow‐up time was 2887 months with an average of 11.7 months. Using multivariate Cox proportional hazards models to adjust for demographic characteristics, there was no statistically significant difference in the VTE recurrence among morbidly obese patients treated with a DOAC compared with VKA (HR: 0.28, 95% CI: 0.07‐1.11, *p* = 0.07) (Table 2). At 1 year, 97 patients (39.3%) were still on anticoagulants (ACs), and 6 (2.4%) were still on ACs at 5 years. The cumulative incidence of recurrent VTE at 6 months, 1 year, and 5 years for patients on VKA was 3.31% (95% CI, 0.55‐10.06), 5.08% (95% CI, 0.43‐59.71), and 3.59% (95% CI, 0.73‐17.63), respectively. Whereas for patients on DOAC, the cumulative incidence of recurrent VTE at 6 months, 1 year, and 5 years was 0.30% (95% CI, 0.05‐1.83), 0.19% (95% CI, 0.02‐2.31), and 0.28% (95% CI, 0.06‐1.37), respectively. The HR for developing recurrent VTE in patients on DOAC versus VKA was 0.30, 0.20, and 0.28, at 6 months, 1 year, and 5 years, respectively (Table 3). The risk of recurrent VTE was not statistically significantly different between DOAC and VKA treatment among morbidly obese patients, *p *= 0.07 (Figure 2). In patients with super obesity, there were no recurrences among those treated with DOACs (*n* = 40) and 2 recurrences occurred among those treated with VKA (*n* = 36).

**TABLE 2:** Multivariate‐adjusted hazard ratios for recurrent venous thromboembolism (VTE) among morbidly obese patients, *n* = 247

**Covariates**	**Hazard ratio**	**95% CI**	***p* Value**
Treatment type			
DOAC	0.279	0.070‐1.110	0.070
VKA	REF		
BMI	0.990	0.923‐1.063	0.789
Age at diagnosis	0.981	0.938‐1.026	0.408
Gender			
Male	2.549	0.776‐8.376	0.123
Female	REF		
Race			
White	1.998	0.625‐6.394	0.243
Non‐white	REF		
Charlson Comorbidity Index	1.323	0.947‐1.848	0.101

Abbreviations: BMI, body mass index; CI, confidence interval; DOAC, direct acting anticoagulant; VKA, vitamin K antagonist.

**TABLE 3:** Hazard ratios (HR) and 95% confidence interval (CI) of recurrent venous thromboembolism (VTE) morbidly obese patients treated with direct acting anticoagulant (DOAC) versus vitamin K antagonist (VKA) at 6 months, 12 months, and 5 years

	**Treatment type**	
**Time**	**HR**	**95% CI**
6 months	0.30	0.05‐1.83
12 months	0.20	0.02‐2.31
5 years	0.28	0.06‐1.37

## 4 DISCUSSION

The present study found that patients with morbid obesity (average BMI 48.2 kg/m^2^) treated with VKAs had a slightly higher risk of recurrence compared to those on DOAC, but this difference was not statistically significant. There were no statistically significant differences in age, BMI, gender, race, or comorbidities when we compared the study population by treatment type, which suggested that, in current routine clinical practice, the patient's BMI does not influence medical decision making related to choice of AC. In this study, we observed the cumulative incidence of recurrence at 5 years to be 0.28% for morbidly obese patients on DOACs and 3.59% for those on VKA. The general hazard of VTE is highest within the first 6 months and patients are always at risk for recurrence regardless of time since the incident VTE event, which aligned with this study, however, the findings were not statistically significant [14]. In clinical trials of DOACs for extended anticoagulation, recurrence rates at 12 months were 1.2%‐1.7% on VKA 0.7% [15, 16]. Recurrence rates in this study population were lower than expected for those on DOACs and higher for those on VKA, though within the CIs of this study population.

Our findings are similar to several other recent retrospective studies. In a study of morbidly obese patients with VTE treated with oral ACs (*n* = 366), a similar risk of recurrence for patients treated with apixaban, rivaroxaban, or VKA was observed [17]. Importantly, their cohort consisted of 25% of patients with a BMI > 50 kg/m^2^. In the presently reported study, approximately 30% of patients had a BMI > 50 kg/m^2^. A retrospective single‐center study of obese patients (BMI > 30 kg/m^2^) by Coons et al. reported similar rates of VTE recurrence between those treated with DOAC compared with VKA [18]. However, only 28.5% of the study subjects were morbidly obese, and this population was not analyzed separately. Furthermore, the proportion of patients with BMI > 50 kg/m^2^ was not reported. In a retrospective study of claims data comparing VTE recurrence among morbidly obese patients treated with rivaroxaban versus warfarin, no difference in rates of VTE recurrence were seen [9]; however, the proportion of patients with BMI > 50 kg/m^2^ in this study is unknown.

Due to the retrospective nature of the study, confounding variables and selection bias were the main limitations. Confounding factors that may have influenced VTE recurrence may include previous surgeries, related genetic disorders, comorbidity, and presence of thrombophilia. However, they were not accounted for in our time‐to‐event analysis due to inadequate data. Additionally, VTE recurrence was determined using medical record review, and thus we may not capture patients who had a recurrence but were not seen within the Emory Healthcare system. Thus, selection bias may have been present. However, the application of this bias would likely be equal among patients receiving DOAC and VKA, thus having little impact on our conclusions drawn from between group comparisons. Finally, this study was a single‐center cohort study, and thus had a smaller dataset and a lack of matching. CIs lacked precision, likely due to the small sample size. Taking everything into consideration, the data showed that the risks of recurrent VTE between DOAC versus VKA were similar among morbidly obese patients.

Our data provides evidence of comparable efficacy of DOAC and VKE in morbidly obese patients with VTE. The hazard for developing recurrent VTE in patients on DOAC versus VKA was 0.30 (95% CI: 0.05‐1.83) at 6 months is reassuring and suggests that the treatment of VTE with DOACs in morbidly obese patients has similar efficacy as VKA. Furthermore, the use of a DOAC eliminates the need for international normalized ratio (INR) monitoring and drug–drug and drug–food interactions simplify therapy. This study, in combination with prior literature, and the robust representation of patients with BMI ≥ 50 kg/m^2^ provides added confidence that DOAC can be safely used for VTE treatment and ongoing prevention in patients with a BMI ≥ 40 kg/m^2^.

## CONFLICT OF INTEREST

The authors declare no conflicts of interest. No funding was received in support of this study.

## AUTHOR CONTRIBUTIONS

The authors confirm contribution to the paper as follows: study conception and design: Juan Li, Christine L Kempton; data acquisition: Juan Li, Christine L Kempton; analysis and interpretation of results: Juan Li, Lia C. Scott, Lorraine A. Cafuir, Manila Gaddh, Christine L Kempton; draft manuscript preparation: Juan Li, Lia C. Scott, Lorraine A. Cafuir, Manila Gaddh, Christine L Kempton. All authors reviewed and approved final version of manuscript.

## Data Availability

De‐identified data can be shared upon request.

## References

[1] Mayer F , Kirchmayer U , Coletta P , Agabiti N , Belleudi V , Cappai G , et al. Safety and effectiveness of direct oral anticoagulants versus vitamin k antagonists: pilot implementation of a near‐real‐time monitoring program in italy. J Am Heart Assoc. 2022;7(6):e008034. 10.1161/JAHA.117.008034 PMC5907561 29525786

[2] Ageno W , Gallus AS , Wittkowsky A , Crowther M , Hylek EM , Palareti G . Oral anticoagulant therapy: antithrombotic therapy and prevention of thrombosis, 9th ed: American College of Chest Physicians Evidence‐Based Clinical Practice Guidelines. Chest. 2012;141(2):e44S‐88S. 10.1378/chest.11-2292 22315269 PMC3278051

[3] Undas A , Góralczyk T . Direct oral anticoagulants in patients with thrombophilia: challenges in diagnostic evaluation and treatment. Adv Clin Exp Med. 2016;25(6):1321‐30. doi:10.17219/acem/65853 28028988

[4] Weitz JI , Jaffer IH . Optimizing the safety of treatment for venous thromboembolism in the era of direct oral anticoagulants. Pol Arch Med Wewn. 2016;126(9):688‐96. doi:10.20452/pamw.3547 27592622

[5] Yang G , De Staercke C , Hooper WC . The effects of obesity on venous thromboembolism: a review. Open J Prev Med. 2012;2(4):499‐509. 10.4236/ojpm.2012.24069 26236563 PMC4520798

[6] Jimenez S , Ruiz‐Artacho P , Merlo M , Suero C , Antolin A , Casal JR , et al. Risk profile, management, and outcomes of patients with venous thromboembolism attended in Spanish Emergency Departments: The ESPHERIA registry. Medicine (Baltimore). 2017;96(48):e8796. 10.1097/MD.0000000000008796 29310357 PMC5728758

[7] Hales CM , Carroll MD , Fryar CD , Ogden CL . Prevalence of obesity and severe obesity among adults: United States, 2017–2018. NCHS Data Brief. 2020;360):1‐8. 32487284

[8] Martin K , Beyer‐Westendorf J , Davidson BL , Huisman MV , Sandset PM , Moll S . Use of the direct oral anticoagulants in obese patients: guidance from the SSC of the ISTH. J Thromb Haemost. 2016;14(6):1308‐13. 10.1111/jth.13323 27299806 PMC4936273

[9] Spyropoulos AC , Ashton V , Chen YW , Wu B , Peterson ED . Rivaroxaban versus warfarin treatment among morbidly obese patients with venous thromboembolism: Comparative effectiveness, safety, and costs. Thromb Res. 2019;182:159‐66. 10.1016/j.thromres.2019.08.021 31493618

[10] Martin KA , Beyer‐Westendorf J , Davidson BL , Huisman MV , Sandset PM , Moll S . Use of direct oral anticoagulants in patients with obesity for treatment and prevention of venous thromboembolism: Updated communication from the ISTH SSC Subcommittee on Control of Anticoagulation. J Thromb Haemost. 2021;19(8):1874‐82. 10.1111/jth.15358 34259389

[11] Quan H , Li B , Couris CM , Fushimi K , Graham P , Hider P , et al. Updating and validating the Charlson comorbidity index and score for risk adjustment in hospital discharge abstracts using data from 6 countries. Am J Epidemiol. 2011;173(6):676‐82. 10.1093/aje/kwq433 21330339

[12] Charlson ME , Pompei P , Ales KL , MacKenzie CR . A new method of classifying prognostic comorbidity in longitudinal studies: development and validation. J Chronic Dis. 1987;40(5):373‐83. 10.1016/0021-9681(87)90171-8 3558716

[13] Fine JP , Gray RJ . A proportional hazards model for the subdistribution of a competing risk. J Am Statist Assoc. 1999;94(446):496–509.

[14] Heit JA . Predicting the risk of venous thromboembolism recurrence. Am J Hematol. 2012;87(1):S63‐7. 10.1002/ajh.23128 22367958 PMC3383031

[15] Agnelli G , Buller HR , Cohen A , Curto M , Gallus AS , Johnson M , et al. Apixaban for extended treatment of venous thromboembolism. N Engl J Med. 2013;368(8):699‐708. 10.1056/NEJMoa1207541 23216615

[16] Weitz JI , Lensing AWA , Prins MH , Bauersachs R , Beyer‐Westendorf J , Bounameaux H , et al. Rivaroxaban or aspirin for extended treatment of venous thromboembolism. N Engl J Med. 2017;376(13):1211‐22. 10.1056/NEJMoa1700518 28316279

[17] Kushnir M , Choi Y , Eisenberg R , Rao D , Tolu S , Gao J , et al. Efficacy and safety of direct oral factor Xa inhibitors compared with warfarin in patients with morbid obesity: a single‐centre, retrospective analysis of chart data. Lancet Haematol. 2019;6(7):e359‐65. 10.1016/S2352-3026(19)30086-9 31133411

[18] Coons JC , Albert L , Bejjani A , Iasella CJ . Effectiveness and safety of direct oral anticoagulants versus warfarin in obese patients with acute venous thromboembolism [published correction appears in Pharmacotherapy. 2020 Jul;40(7):718]. Pharmacotherapy. 2020;40(3):204‐10. 10.1002/phar.2369 31968126

